# Exhaustive assignment of compositional bias reveals universally prevalent biased regions: analysis of functional associations in human and *Drosophila*

**DOI:** 10.1186/1471-2105-7-441

**Published:** 2006-10-10

**Authors:** Paul M Harrison

**Affiliations:** 1 Dept. of Biology, McGill University, Stewart Biology Building, 1205 Dr. Penfield Ave., Montreal, QC, H3A 1B1, Canada

## Abstract

**Background:**

Compositionally biased (CB) regions are stretches in protein sequences made from mainly a distinct subset of amino acid residues; such regions are frequently associated with a structural role in the cell, or with protein disorder.

**Results:**

We derived a procedure for the exhaustive assignment and classification of CB regions, and have applied it to thirteen metazoan proteomes. Sequences are initially scanned for the lowest-probability subsequences (LPSs) for single amino-acid types; subsequently, an exhaustive search for lowest probability subsequences (LPSs) for multiple residue types is performed iteratively until convergence, to define CB region boundaries. We analysed > 40,000 CB regions with > 20 million residues; strikingly, nine single-/double- residue biases are universally abundant, and are consistently highly ranked across both vertebrates and invertebrates. To home in subpopulations of CB regions of interest in human and *D. melanogaster*, we analysed CB region lengths, conservation, inferred functional categories and predicted protein disorder, and filtered for coiled coils and protein structures. In particular, we found that some of the universally abundant CB regions have significant associations to transcription and nuclear localization in Human and *Drosophila*, and are also predicted to be moderately or highly disordered. Focussing on Q-based biased regions, we found that these regions are typically only well conserved within mammals (appearing in 60–80% of orthologs), with shorter human transcription-related CB regions being unconserved outside of mammals; they are also preferentially linked to protein domains such as the *homeodomain *and *glucocorticoid-receptor DNA-binding domain*. In general, only ~40–50% of residues in these human and *Drosophila *CB regions have predicted protein disorder.

**Conclusion:**

This data is of use for the further functional characterization of genes, and for structural genomics initiatives.

## Background

Compositional bias for a subset of residues is a widespread phenomenon in protein sequences; it has historically been linked to proteins having a structural role, or displaying some intrinsic protein disorder [1-3]. Many types of compositionally-biased (CB) region are masked as low-complexity sequence during protein sequence alignment, as a matter of course [4-8], since failure to mask such sequences can lead to a false assumption of evolutionary relatedness. The most commonly used of these masking programs, SEG [7], assesses sequence entropy using user-defined input parameters determining the granularity of the sequence masking.

Previous analysis of compositional bias has focused on single-residue biases, and homopolymeric runs [9-11]. Algorithms that can derive CB regions for multiple residue types have also been developed [6,8]. Here, for the first time, we have derived an exhaustive assignment of CB regions made from multiple residues types, in complete proteomes, substantially developing and expanding the scope of our bias analysis algorithm [6]. The present concept of compositional bias has been developed to enable the assignment and exhaustive analysis of biases for multiple residue types, built up from an initial detection of single-residue biases, in a way that is independent of window-lengths, or similar user-defined parameters. We find that a short list of biases is universally abundant in the metazoan proteomes examined, along with some notable relative species-specific abundances. For human and fruitfly, CB regions are analysed for conservation, length, functional linkages, and predicted protein disorder content. Some of the universally abundant biases are linked to *nuclear localization *and *transcription *in Human and/or *Drosophila*.

## Results & discussion

### Some biases are universally abundant in metazoans

Over 40,000 CB regions in thirteen metazoan proteomes were assigned using the procedures described in *Methods*. Briefly, protein sequences are initially scanned for the lowest-probability subsequences (LPSs) for single amino-acid types; subsequently, an exhaustive search for lowest probability subsequences (LPSs) for multiple residue types is performed iteratively until convergence, to define CB region boundaries. A CB region is labelled with a CB signature (denoted {*abc*...} where ***a***, ***b***, ***c***, ... are the residue types that it comprises, in decreasing order of significance). Each CB region has an associated **P**_min _value. Any region with an initial strong bias for residue type ***a***, and any number of other subsidiary biases is denoted {*a(X)*_n_}. It is important to note that these P-values are only meaningful in a relative sense; the process of probability minimization provides a way to define boundaries for regions comprising complex compositional biases, that are distributed or mingled over the length of a particular subsequence.

What are the most consistently abundant biases across all of the metazoan proteomes? To answer this question, for each proteome, each bias type was ranked in decreasing order of abundance. Then, across all of the proteomes, the mean of this ranking was calculated, as well as the number of times the bias types occurred in the top ten of rankings. The twenty-five bias types with the smallest mean ranking values are listed in Table 1. Strikingly, nine single- and double-residue biases are consistently highly ranked in these proteomes: {C}, {P}, {GP}, {Q}, {ED}, {G}, {E}, {S}, {H} and {T} occur in the top ten of at least six species, both vertebrate and invertebrate (Tables 1 and 2).

**Table 1:** Universally abundant compositional biases ***

**Bias**	Mean rank *	Number of times in top ten **
{C}	1.8	13 (13)
{P}	2.5	13 (13)
{GP}	5.0	12 (13)
{Q}	6.5	11 (13)
{G}	6.9	11 (13)
{E}	8.8	11 (13)
{S}	11.5	11 (13)
{ED}	15.4	6 (12)
{H}	23.7	1 (13)
{RS}	26.8	1 (13)
{T}	31.5	6 (13)
{A}	32.2	3 (13)
{KE}	34.9	0 (13)
{K}	37.6	3 (13)
{SR}	44.6	0 (13)
{QP}	45.6	3 (13)
{R}	52.5	1 (13)
{PA}	53.9	0 (12)
{PG}	56.8	3 (13)
{PM}	56.9	0 (12)
{EQKL}	61.2	1 (9)
{QH}	65.9	2 (13)
{CD}	68.2	1 (13)
{GR}	69.5	0 (13)
{SP}	71.8	0 (10)

* Mean rank is simply calculated from averaging over rankings (in decreasing order of abundance) for all thirteen proteomes.

** Number of times the bias appears in the top 10 (with the number of proteomes this bias occurs in, in brackets).

*** The types of CB region have been ranked in increasing order of mean rank for the human proteome.

**Table 2:** Top biases for the the thirteen metazoan proteomes (*)

**Mammals**															
**Hsap**		**Ptro**		**Mmus**		**Rnor**									

{C}	0.036	{GP}	0.042	{C}	0.039	{C}	0.039								
{P}	0.031	{C}	0.037	{P}	0.020	{GP}	0.023								
{GP}	0.024	{P}	0.020	{GP}	0.020	{P}	0.020								
{Q}	0.009	{ED}	0.009	{Q}	0.011	{Q}	0.013								
{G}	0.008	{Q}	0.009	{ED}	0.009	{ED}	0.009								
{E}	0.008	{G}	0.009	{E}	0.008	{KE}	0.006								
{S}	0.008	{S}	0.007	{PQ}	0.005	{E}	0.005								
{ED}	0.007	{E}	0.007	{CG}	0.005	{RS}	0.005								
{PG}	0.007	{QP}	0.007	{PG}	0.004	{S}	0.004								
{QP}	0.006	{PG}	0.006	{G}	0.004	{PG}	0.004								

**Total**	**4903**	**Total**	**3812**	**Total**	**3721**	**Total**	**3169**								

**Non-mammals**															

**Ggal**		**Frub**		**Tnig**		**Drer**		**Agam**		**Amel**		**Dmel**		**Cele**	

{C}	0.056	{HT}	0.099	{C}	0.034	{C}	0.042	{C}	0.035	{C}	0.052	{Q}	0.070	{GP}	0.035
{GP}	0.048	{CV}	0.081	{GP}	0.032	{GP}	0.038	{GP}	0.014	{GP}	0.030	{QH}	0.055	{C}	0.030
{P}	0.019	{GP}	0.048	{P}	0.016	{P}	0.017	{T}	0.012	{P}	0.016	{C}	0.020	{T}	0.021
{EKQL}	0.008	{C}	0.046	{CV}	0.014	{T}	0.010	{Q}	0.012	{F}	0.010	{T}	0.014	{Q}	0.012
{Q}	0.007	{P}	0.016	{HT}	0.013	{ED}	0.010	{QH}	0.009	{R}	0.007	{N}	0.011	{KED}	0.010
{S}	0.006	{Q}	0.009	{Q}	0.007	{G}	0.008	{G}	0.009	{G}	0.007	{H}	0.009	{QC}	0.009
{EKQ}	0.006	{S}	0.009	{PS}	0.007	{Q}	0.007	{RDE}	0.007	{FIY}	0.007	{S}	0.007	{ED}	0.009
{RS}	0.005	{CD}	0.008	{ED}	0.007	{S}	0.007	{PIE}	0.007	{CN}	0.007	{G}	0.006	{KE}	0.008
{QP}	0.005	{E}	0.008	{E}	0.006	{RS}	0.005	{P}	0.005	{EKAQ LRND}	0.006	{QPH}	0.006	{PG}	0.007
{EQKL}	0.005	{ED}	0.007	{PG}	0.006	{HC}	0.005	{RS}	0.005	{A}	0.005	{P}	0.006	{RS}	0.007

**Total**	**2743**	**Total**	**5639**	**Total**	**2609**	**Total**	**3669**	**Total**	**1304**	**Total**	**2340**	**Total**	**3394**	**Total**	**2295**

(*) The proteomes are given an abbreviation derived from the Latin name of the organism, *i.e*., *Hsap *for human, *Rnor *for rat, *Drer *for zebrafish, *etc*. The *Total Number *of CB regions is listed at the bottom for each proteome. For each bias (denoted by a CB signature), the fraction of the total number of CB regions is given; the regions are listed in decreasing order of abundance.

Some abundant species-specific biases stand out, *e.g*., {Q} regions are most abundant in the fruitfly (Table 2), when compared to all the other proteomes, and, in combination with {QH} regions (the second most prevalent bias in fruitfly) and {QPH} regions, comprise 13% of all the CB regions in that organism. These CB regions will be discussed in more detail below.

Other examples of abundant species-specific biases may be indicative of spurious gene predictions. Examination of examples of the many {HT} and {CV} regions found in the two puffer-fish proteomes (Table 2), indicates that they arise from genome regions with simple repeats, and typically have poorly predicted introns; these thus may arise from systematic errors in gene prediction.

**Figure 1:**
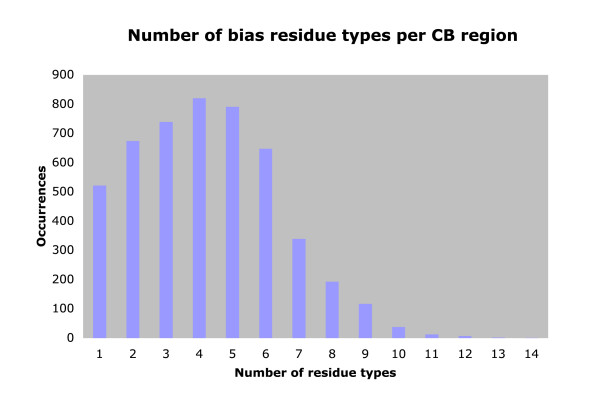
Number of bias residue types per CB region in the human proteome. The number of bias residue types per CB region is binned in a bar chart (x-axis). The total occurrences for each 'number of bias residue types' is on the y-axis.

Although many of the most abundant biases across the metazoans are made from either one or two residue types, most biased regions are comprised of a larger number of residues, with a broad mode from about 3 to 5 residue types. This is illustrated for the human proteome (Figure 1). More than a quarter (~27%) of the human CB regions have signatures of ≥ 6 residue types; this is because the bias assignment algorithm can detect CB regions that are composed of multiple milder single-residue biases. (An example of such a region is given in Figure 7(C) below.)

### Functional biases and predicted protein disorder content of the top ten biases in human and *Drosophila*

Obviously, these bias prevalences represent many diverse types of protein subsequence; therefore, to pick out specific subpopulations that are of interest, we need to perform some further characterizations. To this end, for the CB regions in both the human and *Drosophila *proteomes, after filtering for coiled coils and known protein structures, we examined: *(i) *significant functional associations based on Gene Ontology (GO) categories and terms; *(ii) *predicted protein disorder content (using the program DISOPRED [12]); *(iii) *CB region length; *(iv) *CB region conservation. We focus specifically on Q-based and E-based biases, as specific examples.

Tables 3 and 4 show that most of the top ten biases (6/10 for both human and *Drosophila) *come from the 'universally prevalent' list; some of these have significant associations with *transcriptional *functional categories and with *nuclear *localization. These CB regions also have moderate to high predicted protein disorder contents (***D ***value ~0.4–0.8) (Tables 3 and 4). The ***D ***value is the fraction of the CB region that is predicted to be disordered by the program DISOPRED [12].

**Table 3:** Most abundant CB regions in Human and their significant functional associations and predicted protein disorder (*)

**Bias**	**Number of members**	**Mean disorder (D) value**	**Functional categories** (GO term [# of occurrences]; description; *P' *value)
**{P}**	273	0.61	GO:0005737 [55]; cytoplasm (3 × 10^-23^)
			GO:0007155 [28]; cell adhesion (4 × 10^-10^)
**{C}**	183	0.00	GO:0005515 [37]; protein-binding (2 × 10^-4^)
			GO:0005509 [35]; calcium-ion binding (3 × 10^-15^)
			GO:0007155 [29]; cell adhesion (5 × 10^-16^)
			GO:0005198 [27]; structural-molecule activity (4 × 10^-16^)
			GO:0046872 [21]; metal-ion binding (4 × 10^-3^)
			GO:0005578 [16]; extracellular matrix (sensu Metazoa) (8 × 10^-9^)
**{GP}**	116	0.76	GO:0005737 [65]; cytoplasm (2 × 10^-61^)
			GO:0007155 [27]; cell adhesion (2 × 10^-19^)
			GO:0005198 [12]; structural-molecule activity (2 × 10^-3^)
			GO:0005578 [9]; extracellular matrix (sensu Metazoa) (8 × 10^-3^)
**{Q} **^†^	77	0.41	GO:0005634 [34]; nucleus (3 × 10^-8^)
			GO:0006355 [21]; DNA-dependent regulation of transcription (1 × 10^-4^)
**{S}**	74	0.71	
**{G}**	70	0.40	GO:0005634 [24]; nucleus (2 × 10^-2^)
**{E}**	69	0.49	GO:0005198 [10]; structural-molecule activity (1 × 10^-3^)
**{ED} ^†^**	33	0.56	GO:0005634 [24]; nucleus (1 × 10^-11^)
			GO:0006355 [14]; DNA-dependent regulation of transcription (6 × 10^-5^)
**{PG}**	32	0.75	
**{QP} ^†^**	30	0.52	

(*) – The CB regions are sorted in decreasing order of abundance. They are denoted by their CB signatures in *column #1*. *Column #2 *contains the total number of members in a particular cluster; *column #3 *is the mean value of *D*, the disorder fraction of each member; *column #4 *lists the top five significantly-associated (P' ≤ 0.05, adjusted for multiple hypothesis testing) GO (Gene Ontology terms for the cluster in the format: *GO term name *[count for GO term]; *description of GO term in words*. In addition, bias types that are significantly associated with transcription (where we reduced GO categories to just two categories, '*transcription-related*' and '*non-transcription-related'*), are labelled with a **† **sign.

**Table 4:** Top Ten Biases for Fruitfly, and their significant functional associations and protein disorder values (*)

**Bias**	**Number of members**	**Mean disorder (D) value**	**Functional categories** (GO term [# of occurrences]; description; *P' *value)
**{Q} ^†^**	274	0.45	GO:0005634 [78]; nucleus (2 × 10^-14^)
			GO:0006357 [53]; regulation of transcription from RNA polymerase II promoter (1 × 10^-16^)
			GO:0003700 [44]; transcription factor activity (3 × 10^-12^)
			GO:0003677 [37]; DNA binding (7 × 10^-6^)
			GO:0005515 [33]; protein binding (8 × 10^-3^)
			GO:0003704 [20]; specific RNA polymerase II transcription factor activity (2 × 10^-5^)
**{QH} ^†^**	187	0.81	GO:0005634 [75]; nucleus (2 × 10^-24^)
			GO:0003700 [52]; transcription factor activity (3 × 10^-27^)
			GO:0006357 [45]; regulation of transcription from RNA polymerase II promoter (9 × 10^-18^)
			GO:0008270 [36]; Zn-ion binding (9 × 10^-12^)
			GO:0003677 [35]; DNA binding (1 × 10^-9^)
			GO:0006355 [30]; DNA-dept. regulation of transcription (3 × 10^-13^)
			GO:0003702 [29]; RNA polymerase II transcription factor activity (4 × 10^-16^)
			GO:0045449 [26]; regulation of transcription (6 × 10^-15^)
			GO:0007498 [22]; mesoderm development (1 × 10^-7^)
**{C}**	70	0.00	GO:0005198 [25]; structural molecule activity (2 × 10^-27^)
			GO:0007165 [23]; signal transduction (5 × 10^-14^)
			GO:0016337 [19]; cell-cell adhesion (1 × 10^-19^)
			GO:0005886 [14]; plasma membrane (1 × 10^-3^)
			GO:0005102 [14]; receptor binding (6 × 10^-9^)
**{P}**	62	0.13	
**{T}**	61	0.28	
**{N}**	58	0.45	GO:0005634 [19]; nucleus (3 × 10^-2^)
			GO:0003729 [16]; mRNA binding (1 × 10^-7^)
			GO:0003723 [16]; RNA binding (1 × 10^-11^)
**{G}**	50	0.14	
**{H}**	44	0.42	
**{S}**	38	0.25	
**{A} ^†^**	38	0.21	GO:0005634 [16] nucleus (3 × 10^-3^)
			GO:0006357 [14]; regulation of transcription from RNA polymerase II promoter (3 × 10^-6^)
			GO:0006333 [11]; chromatin (dis)assembly (4 × 10^-10^)
			GO:0003700 [10]; transcription factor activity (2 × 10^-2^)

(*) – The CB regions are sorted in decreasing order of abundance. They are denoted by their CB signatures in *column #1*. *Column #2 *contains the total number of members in a particular cluster; *column #3 *is the mean value of *D*, the disorder fraction of each member; *column #4 *lists the top five significantly-associated (P' ≤ 0.05) GO (Gene Ontology terms for the cluster in the format: *GO term name *[count for GO term]; *description of GO term in words*. In addition, bias types that are significantly associated with transcription (where we reduced GO categories to just two categories, '*transcription-related' *and '*non-transcription-related'*), are labelled with a **† **sign.

For example, {ED} regions in human have significant associations to 'nucleus' and 'DNA-dependent regulation of transcription', and are on average predicted to be moderately disordered (mean ***D ***values of 0.56) (Table 3). {Q} regions (in both *Drosophila *and human) and {QH} regions (in *Drosophila *only) have similar functional associations, and are predicted to be moderately to highly disordered (***D ***~0.4–0.8) (Tables 3 and 4).

Additionally, we separated GO terms into those that are transcription-associated and those that are not (see *Methods *for details). Then, using these two 'supercategories', we tested for significant association with the transcription supercategory for each CB region type. For both human and *Drosophila*, the CB regions that demonstrate such a significant association with the transcription supercategory, also have significant association to individual GO terms linked to transcription (Tables 3 and 4).

### Further analysis of nuclear-/transcription-related biases

#### GO and protein domain associations for the largest CB region grouping, {Q(X)_n_}

Since {Q} regions, and {Q(X)_n_} in general, represent the most numerous CB region grouping in either human or *Drosophila*, we examined the top twenty significant GO assignments for {Q(X)_n_} regions in more detail for *Drosophila *and Human, as well as for Rat and Mouse (Table 5). Noticeably, across *Drosophila *and the three mammals, 'DNA-dependent regulation of transcription', 'transcription factor activity' and 'nucleus' are all highly-ranked functional associations. Similar prevalences are observed for abundant GO terms, if all {Q}+{QH}+{QPH} regions are analyzed in the same way (not shown).

**Table 5:** Most abundant GO terms for {Q(X)_n_} CB regions in the fruitfly, mouse, rat and human proteomes *

**Fruitfly (total = 835)**		**Rat (total = 234)**		**Mouse (total = 267)**		**Human (total = 335)**	
Number**	**GO term and its description**	Number**	**GO term and its description**	Number**	**GO term and its description**	Number**	**GO term and its description**

245	**GO:0005634 ; nucleus**	38	**GO:0005634 ; nucleus**	78	**GO:0005634 ; nucleus**	114	**GO:0005634 ; nucleus**
152	*GO:0006357 ; regulation of transcription from RNA polymerase II promoter*	28	**GO:0006355 ; DNA-dependent regulation of transcription**	49	**GO:0006355 ; DNA-dependent regulation of transcription**	68	**GO:0006355 ; DNA-dependent regulation of transcription**
137	**GO:0003700 ; transcription factor activity**	15	**GO:0003700 ; transcription factor activity**	36	GO:0005515 ; protein-binding	51	GO:0008270 ; Zinc ion binding
125	*GO:0003677 ; DNA-binding*	6	GO:0004871 ; signal transducer activity	31	*GO:0003677 ; DNA-binding*	39	**GO:0003700 ; transcription factor activity**
99	GO:0005515 ; protein-binding	4	GO:0030216 ; keratinocyte differentiation	28	GO:0008270 ; Zinc ion binding	35	*GO:0003677 ; DNA-binding*
92	GO:0008270 ; Zinc ion binding	4	GO:0001533 ; cornified envelope	25	**GO:0003700 ; transcription factor activity**	24	*GO:0003676 ; nucleic acid binding*
78	**GO:0006355 ; DNA-dependent regulation of transcription**			21	GO:0005737 ; cytoplasm	21	GO:0046872 ; metal-ion binding
62	GO:0005737 ; cytoplasm			12	*GO:0006350 ; transcription*	20	*GO:0003713 ; transcriptional coactivator activity*
61	GO:0007498 ; mesoderm development			*9*	*GO:0045944 ; positive regulation of transcription from RNA pol II promoter*	17	*GO:0006350 ; transcription*
59	*GO:0003677 ; RNA polymerase II transcription factor activity*			9	*GO:0003713 ; transcription coactivator activity*	11	*GO:0006366 ; transcription from RNA pol II promoter*
57	*GO:0003729 ; mRNA binding*			5	*GO:00016564 ; transcriptional repressor activity*	11	GO:0004871 ; signal transducer activity
53	*GO:0045449 ; regulation of transcription*			5	*GO:00016563 ; transcriptional activator activity*	10	*GO:00016563 ; transcriptional activator activity*
47	GO:0009993 ; oogenesis (sensu insecta)					8	*GO:0003702 ; RNA pol II transcription factor activity*
47	GO:0007398 ; ectoderm development					6	*GO:0006367 ; Transcription initiation from RNA pol II promoter*
47	*GO:0003704 ; specific RNA polymerase II transcription factor activity*						
43	*GO:0030528 ; transcription regulatory activity*						
41	GO:0008283 ; cell proliferation						
36	GO:0003779 ; actin binding						
32	GO:0007476 ; wing morphogenesis						
30	GO:0007242 ; intracellular signaling cascade						

* GO terms common to all four organisms are in **bold**. Other terms directly associated with 'transcription' or 'nucleic acids' are in *italics*.

** Number of occurrences of each GO term. For each proteome, the GO terms are sorted in decreasing order of abundance.

The {Q(X)_n_} grouping is also sufficiently numerous that we can count up the most frequently associated globular domains (*i.e*., domains that are in the same sequences) (Table 6). The most commonly associated domain in both Human and Drosophila is the 'DNA/RNA-binding three-helical bundle', chiefly arising from the 'Homeodomain-like' superfamily. This domain was first found in *Drosophila *homeotic genes, and occurs widely in transcription factors; related domains are also used in other DNA-binding proteins, such as telomeric proteins, recombinases, *etc*.

**Table 6:** Associated SCOP domains for *Q{(X)_*n*_} *regions in Human and Fruitfly (*)

**Fruitfly**				**Human**			
Protein folds	Number	Superfamilies	Number	Protein folds	Number	Superfamilies	Number

**a.4, DNA/RNA-binding 3-helical bundle**	53	**a.4.1, Homeodomain-like**	32	**a.4, DNA/RNA-binding 3-helical bundle**	14	g.50.1, FYVE/PHD Zinc finger	14
g.39, glucocorticoid receptor-like (DNA-binding domain)	17	g.39.1, glucocorticoid receptor-like (DNA-binding domain)	17	g.50, FYVE/PHD Zinc finger	11	a.40.1, calponin homology (CH) domain	12
b.1, Ig-like sandwich	16	a.4.5, winged-helix DNA-binding domain	16	a.40, CH-domain -like	9	d.211.2, plakin repeat	10
**d.144, protein kinase -like**	14	**d.144.1, protein kinase -like**	14	d.211, beta-hairpin-alpha-hairpin repeat (ankyrin & plakin)	8	**d.144.1, protein kinase -like**	10
b.34, SH3-like barrel	12	a.123.1, nuclear-receptor ligand-binding domain	12	**d.144, protein kinase -like**	6	**a.4.1, homeodomain-like**	10
a.123, nuclear-receptor ligand-binding domain	12						

(*) For each proteome, the most common SCOP domains **[18] **are listed in decreasing order of abundance. Those that occur in both the Human and Fruitfly lists are highlighted in bold.

#### CB region length

**Figure 2:**
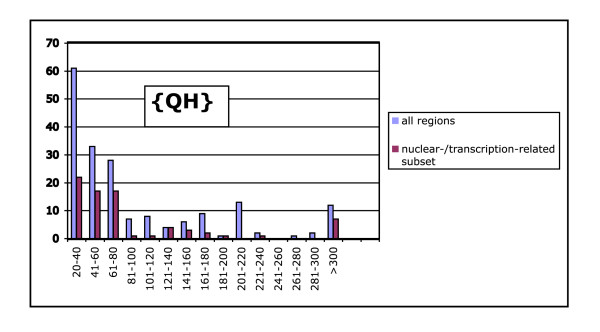
Distribution of lengths of {QH} regions in *D. melanogaster*. There are two histograms: the overall distribution (red bars), and the nuclear- or transcription-related proteins (blue bars). The nuclear- and transcription-related proteins have been compiled by grouping together all proteins that have been assigned one of the GO terms that has been adjudged transcription-related (See main text for details).

**Figure 3:**
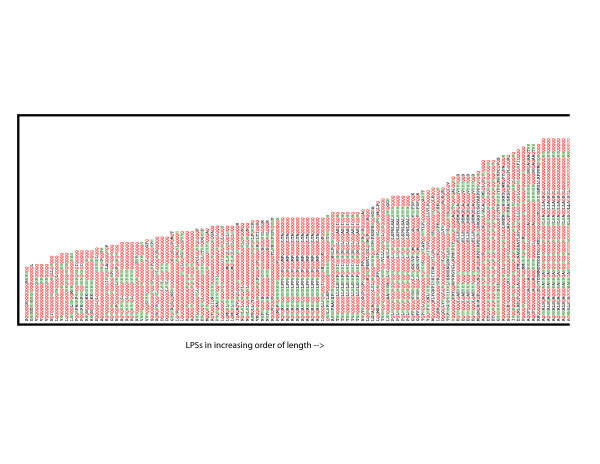
A 'blow-up' of the overall distribution of {QH} region lengths. The {QH} regions are listed horizontally in order of increasing length; Q residues are coloured red and H residues green, with other residues in black.

In general, the nuclear-/transcription-related biases show a mode in region length at 20–40 residues. This is shown specifically for {QH} regions in Figure 2. A similar fall-off is observed for the distribution for the subset of {QH} regions that are labelled in the GO classification as associated with 'transcription' or localization in the 'nucleus'. A 'blow-up' of the overall {QH} histogram (Figure 3) demonstrates that these regions are not adequately analysed simply as homopolymeric tracts. The subsidiary nature of the H component of the bias is evident, as it is interspersed with longer homopolymeric runs of Q.

#### Conservation

**Figure 4:**
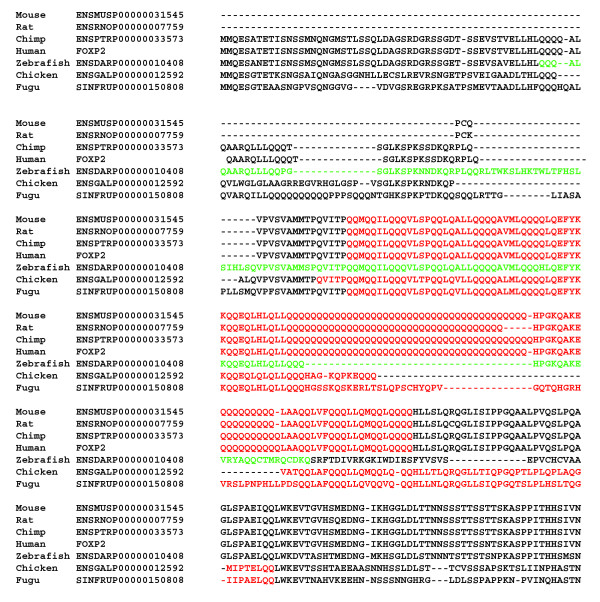
Example of conservation of {Q} region in vertebrates: FOXP2 and its orthologs. A multiple alignment is shown for FOXP2 and its orthologs on other vertebrates, made using the MUSCLE program **[21]**; the {Q} region is highlighted in red if its P-value was high enough to be included in the present analysis; otherwise, it is highlighted in green.

As case studies, we examined the conservation of {Q(X)_n_} and {E(X)_n_} regions in other metazoans, relative to human. Orthologs of proteins were determined with the bi-directional best hits approach, using BLASTP [13] (e-value ≤ 0.0001 with alignment over 0.6 of the length of both sequence, both with and without masking compositionally biased parts). We analysed the fraction of orthologs that maintain a biased region of the same character ({Q(X)_n_} or {E(X)_n_}) (Table 7). Generally, these regions (filtered for coiled coils), show high conservation in orthologs from other mammals (60–80% depending on criteria), and low conservation in invertebrates (0–50%) (Table 7). Obviously, these numbers broadly cover a diverse set of CB regions; visual curation reveals that shorter {Q(X)_n_} and {E(X)_n_} CB regions consisting of short homopolymeric runs of {Q} are not conserved from human to invertebrates, and that all of the regions that are conserved are longer (> ~90 residues). Indeed, this lack of conservation in invertebrates is also evident when one examines specifically the {Q}+{Q}+{QPH} and {ED}+{E} subsets (Table 7). A multiple alignment of FOXP2, a gene important in language in humans, is illustrated as an example of conservation of a {Q} region defined in vertebrate proteomes (Figure 4).

**Table 7:** Conservation of *{Q(X)_*n*_} *and *{E(X)_*n*_} *biased regions (*)

Conservation♦	Total Number	Human ♦ Mouse		Human ♦ Rat		Human ♦ Chicken		Human ♦ C.elegans		Human ♦ Fruitfly		Fruitfly ♦ Human	
Human bias regions		With CB region	W/o CB region	With CB region	W/o CB region	With CB region	W/o CB region	With CB region	W/o CB region	With CB region	W/o CB region	With CB region	W/o CB region
All Q-rich regions {Q(X)_n_}	350	255/326 (78%)	97/140 (69%)	245/315 (78%)	100/135 (74%)	184/281 (65%)	100/160 (63%)	46/115 (40%)	3/18 (17%)	79/255 (31%)	12/36 (33%)	79/246 (32%)	12/13 (92%)
{Q}, {QH} and {QPH} regions	139	73/109 (67%)	41/66 (62%)	61/100 (61%)	38/64 (59%)	30/93 (32%)	25/81 (31%)	1/30 (3%)	0/1 (0%)	11/53 (21%)	0/12 (0%)	16/80 (20%)	0/11 (0%)
All E-/D-rich regions {E/D(X)_n_}	298	194/268 (72%)	107/169 (63%)	184/264 (70%)	96/155 (62%)	125/219 (57%)	72/152 (47%)	50/105 (48%)	14/46 (30%)	66/244 (27%)	17/53 (32%)	66/130 (51%)	13/28 (46%)
{E} and {ED} regions	102	55/89 (62%)	41/62 (66%)	53/89 (60%)	33/59 (56%)	13/62 (21%)	26/83 (31%)	3/32 (9%)	1/17 (6%)	5/40 (13%)	0/12 (0%)	3/49 (6%)	0/16 (0%)

(*) For each bias grouping, the total number of regions is listed, followed by the (total number conserved with the bias region/total number conserved) (percentage in brackets) for each of the proteomes: mouse, rat, chicken, C. elegans and Drosophila (fruitfly), relative to Human. For Drosophila, the 'reverse' conservation is also listed.

#### Predicted protein disorder – general observations

**Figure 5:**
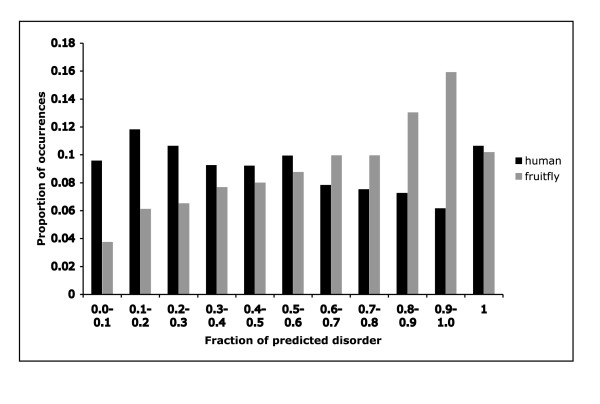
The fraction of predicted disorder (denoted ***D ***in the text) is binned as a bar chart for both the human and fruitfly proteomes. The bin *p-q *contains all values ***D***, such that *p *≤ ***D ***<*q*. The proportion of occurrences in each bin is given on the y-axis.

**Figure 6:**
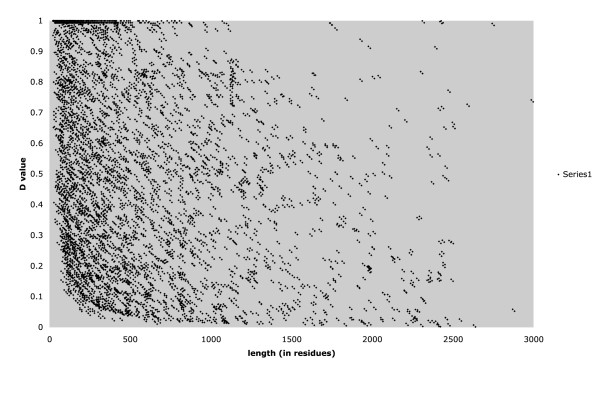
Plot of the ***D ***value versus the length of a CB region for the human proteome.

Prediction of protein disorder has recently been the focus of much research activity [1,12,14]. Such regions present a challenge for further proteome-scale experimental characterization. We analyzed the predicted protein disorder content of the human and *Drosophila *CB regions, using the program DISOPRED [12]. In summed total (simply adding up the total amounts of residues), the human CB region data is predicted to be ~42% disordered, with a similar value observed for the fruitfly (45%). This compares to 17% (human) and 15% (fruitfly) for the whole proteomes of these organisms, indicating a strong relationship between the defined CB regions and predicted protein disorder. However, most predicted protein-disorder is not defined as compositionally biased (67% of predicted protein disorder regions ≥ 20 residues in human, and 72% in fruitfly). Figure 5 shows that distribution of the fraction of disorder (denoted ***D***) predicted for each CB region for human and fruitfly, is approximately uniform; a wide diversity of predicted protein disorder contents is also illustrated by plots of ***D ***versus CB region length (shown for human in Figure 6).

**Figure 7:**
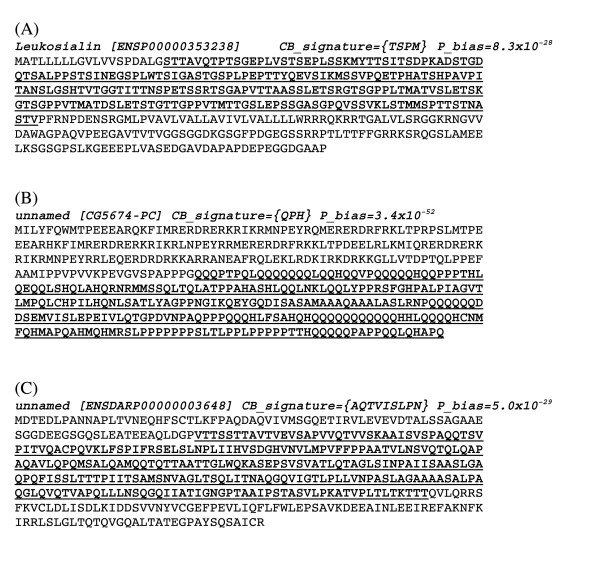
Examples of assigned CB regions. In each case, the name of the protein, its current Ensembl identifier, its CB signature and **P**_min _value are indicated. The CB region is in bold and underlined; the rest of the sequence is in plain text. The proteins are as follows: (A) leukosialin from the human protein, (B) and unnamed fruitfly protein and (C) an unnamed chicken protein.

We examined the inferred cellular compartment for the CB regions, divided into four different groupings according to their ***D ***values, and then calculated propensities to have these compartments for each disorder grouping (Table 8). For human, biased regions have a propensity to be *nuclear *if ***D ***> 0.25, and to be *nuclear *regardless of ***D ***value for the fruitfly. Also, for very high disorder values (***D ***> 0.75), there is significant linkage to both *nuclear *and *cytoplasmic *compartments for both human and fruitfly.

**Table 8:** Cellular compartments for protein with CB regions with different D values (*)

**HUMAN**							
**Overall**	**#**						
**GO:0005634**	**954/4618† (10**^-63^**)**						
Nucleus							
**GO:0005737**	**224/4618† (10**^-5^**)**						
Cytoplasm							
GO:0016020238/4618							
Membrane							
**D ≤ 0.25**	**#**	**0.25 < D ≤ 0.5**	**#**	**0.5 < D ≤ 0.75**	**#**	**D > 0.75**	**#**
GO:0005634	137/980	**GO:0005634**	**206/867† **(10^-18^)	**GO:0005634**	**167/758† **(10^-11^)	**GO:0005634**	**196/948† **(10^-10^)
Nucleus		Nucleus		Nucleus		Nucleus	
GO:0005737	38/980	GO:0005737	35/867	GO:0005737	37/758	**GO:0005737**	**98/948†**(10^-19^)
Cytoplasm		Cytoplasm		Cytoplasm		Cytoplasm	
GO:0016020	68/980	GO:0016020	29/867	GO:0016020	31/758	GO:0016020	34/948
Membrane		Membrane		Membrane		Membrane	
**FRUITFLY**							
**Overall**	**#**						
**GO:0005634**	**593/2972† (10**^-62^**)**						
Nucleus							
GO:0005737	141/2972						
Cytoplasm							
GO:0016020	49/2972						
Membrane							
**D ≤ 0.25**	**#**	**0.25 < D ≤ 0.5**	**#**	**0.5 < D ≤ 0.75**	**#**	**D > 0.75**	**#**
**GO:0005634**	**65/372† **(10^-2^)	**GO:0005634 **	**120/556 † **(10^-14^)	**GO:0005634 **	**168/678† **(10^-27^)	**GO:0005634**	**270/1143† **(10^-10^)
Nucleus		Nucleus		Nucleus		Nucleus	
GO:0005737	20/372	GO:0005737	19/556	GO:0005737	38/678	**GO:0005737 **	**65/1143† **(10^-20^)
Cytoplasm		Cytoplasm		Cytoplasm		Cytoplasm	
GO:0016020	6/372	GO:0016020	12/556	GO:0016020	7/678	GO:0016020	16/1143

(*) The numbers of CB regions (overall, and for four categories split up according to ***D ***value) that have the GO term annotation for Nucleus, Cytoplasm and Membrane are counted up.

**† **Significant overrepresentation using binomial statistics (P' < 0.05), corrected for multiple hypothesis testing over cellular compartment GO terms. P' values are indicated in brackets, rounded up to the nearest power of ten.

## Conclusion

We have derived a method for assignment of compositionally-biased regions and have applied it consistently to the proteomes of thirteen metazoans. We found that a number of biases are universally abundant in metazoans ({P}, {Q}, {GP}, {C} and {ED}), but that there are also some interesting species-specific tendencies, such as the large proportion of {Q}, {QH}, {QHP} and {QPH} regions in the fruitfly proteome. To delineate subpopulations of CB regions of particular interest, we filtered for coiled coils and known protein structures, and examined significant functional associations, predicted protein disorder content (using the program DISOPRED [12]), CB region length, and conservation in Human and *Drosophila*. We found that some of the universally prevalent biases in metazoans are significantly associated with *transcription regulation *and *nuclear localization *in human and/or *Drosophila*. Furthermore, the CB regions identified are not necessarily contiguous with predicted disordered domains (only 40–50% of the residues in these regions are also in predicted disordered regions).

The CB assignment data presented here will be of further use to home in on functional associations. Furthermore, this classification will also help to delineate systematic errors in genome annotation, such as likely false-positive protein motif matches, or subsets of spurious gene predictions (as noted above for the two puffer fish genomes). The CB data can also be used for further characterization of subtypes of protein disorder [15]. It is also useful for informing strategies in structural genomics projects, since such projects rely on the correct parsing of domains and subsequences. Further data relating to the analysis in this paper is available from the author.

## Methods

### Exhaustive assignment of CB regions

The proteomes of thirteen higher eukaryotes were downloaded from the Ensembl website [16], in November 2004. They are [versions in square brackets]: human [build 34], chimpanzee [CHIMP1], mouse [NCBIM33], rat [RGSC3.1], fruit fly [version 3], mosquito (*A. gambiae*) [MOZ2a], honey bee [1^st ^assembly], zebra fish [ZFISH4], and two puffer fish species (*Fugu rubripes *[FUGU2]*, Tetraodon nigriviridis *[TETRAODON7]). The total combined amino-acid composition of all of these proteomes was calculated, and used as the standard for all subsequent calculations. CB assignment was performed using a development of the algorithm previously described for classification of regions with single-residue biases (Harrison and Gerstein, 2003). The assignment of CB regions comprises two steps: *(i) *initial search for single-residue LPSs, and *(ii) *iterative build-up of multiple-residue biases until convergence, *i.e*., until no lower probability subsequence for a given set of bias residues can be found.

#### (i) Initial search for single-residue lowest probability subsequences (LPSs)

We searched for biased regions for each of the 20 amino-acid types as described previously (Harrison and Gerstein, 2003). For each amino-acid type *x*, and for the range of window sizes (20 ≤ *w *≤ 2,500 residues), we search each protein sequence for stretches that have compositional bias of the lowest probability (**P**_min_):

*P*_min _= [*P*_*bias *_(*i, w*)], ∀ i and x     **(1)**

where *i *is each possible start position for a window *w *in the sequence. The probability **P_bias_(*i*,*w*) **in equation (1) is given by a binomial distribution:

Pbias(i,w)=[w!n!(w−n)!]·(fx)n·(1−fx)w−n     (2)
 MathType@MTEF@5@5@+=feaafiart1ev1aaatCvAUfKttLearuWrP9MDH5MBPbIqV92AaeXatLxBI9gBaebbnrfifHhDYfgasaacH8akY=wiFfYdH8Gipec8Eeeu0xXdbba9frFj0=OqFfea0dXdd9vqai=hGuQ8kuc9pgc9s8qqaq=dirpe0xb9q8qiLsFr0=vr0=vr0dc8meaabaqaciaacaGaaeqabaqabeGadaaakeaacqWGqbaudaWgaaWcbaGaemOyaiMaemyAaKMaemyyaeMaem4CamNaeiikaGIaemyAaKMaeiilaWIaem4DaCNaeiykaKcabeaakiabg2da9maadmaabaWaaSaaaeaacqWG3bWDcqGGHaqiaeaacqWGUbGBcqGGHaqidaqadaqaaiabdEha3jabgkHiTiabd6gaUbGaayjkaiaawMcaaiabcgcaHaaaaiaawUfacaGLDbaacqWIpM+zdaqadaqaaiabdAgaMnaaBaaaleaacqWG4baEaeqaaaGccaGLOaGaayzkaaWaaWbaaSqabeaacqWGUbGBaaGccqWIpM+zdaqadaqaaiabigdaXiabgkHiTiabdAgaMnaaBaaaleaacqWG4baEaeqaaaGccaGLOaGaayzkaaWaaWbaaSqabeaacqWG3bWDcqGHsislcqWGUbGBaaGccaWLjaGaaCzcamaabmaabaacbeGae8NmaidacaGLOaGaayzkaaaaaa@5F98@

where *f*_*x *_is the proportion of amino-acid type *x *as given by the total combined composition of all of the proteomes. The count for *x *is denoted *n *in the window *w *starting at position *i*. Sequence stretches with **P**_min _are termed LPSs (*L*owest *P*robability *S*ubsequences), as they have the smallest **P**_bias _values for a given residue type and protein sequence.

#### (ii) Iterative build-up of multiple-residue biases

The procedure described in *(i) *was generalized to calculate biases derived from any number of residue types exhaustively for a given protein sequence, as follows. **P**_min _values are calculated for any set of amino acids {*xyz*...}, by summing up the number of residues over the whole residue-type set; however, they only picked in preference over a previously-calculated bias made by a smaller number of residue types, if their **P**_min _values are smaller. The set of residue types contributing to the bias (sorted in decreasing order of their original **P**_min _values), is defined as the *CB signature*.

The build-up of multiple-residue biases is performed as follows. For each protein sequence, all single-residue LPSs are sorted in decreasing order of **P**_min_. These initial sorted single-residue LPSs thus have a single-letter *CB signature*. Then, iteratively until convergence, for each LPS, the list of LPSs of higher **P**_min _value is searched to check for mutual overlap > 10 residues between the two regions. For all such overlapping pairs, the LPS for the combined residue-type set is calculated, and a new CB signature is derived if the combined **P**_min _is smaller. This procedure is performed iteratively until convergence. Using this procedure, regions that comprise mild bias for multiple residue types can be detected as significantly biased. Three examples of CB regions defined using the above procedure are shown in Figure 7; the first example (A) is a {TPSM} region in leukosialin from the human proteome, the second (B) is a {QPH} region from an un-named protein in the fruitfly, and the third (C) is an un-named protein from chicken which has a {AQTVISLPN} region N-terminal to a POU transcription factor domain. This last example demonstrates how the algorithm can detect a biased region that is composed of many mild, single-residue biases.

### Classification of CB regions

To classify CB regions across a whole proteome, suitable thresholds for **P**_min _must be derived for deciding on inclusion in the analysis. **P**_min _thresholds were derived as follows. Longer protein sequences can have more significantly biased subsequences. To allow for this sequence length -dependent effect, we calculated a sequence length -dependent **P**_min _threshold. For a random sample of 10,000 protein sequences, **P**_min _for the most biased subsequence was plotted against sequence length on a log-log scale. To extract the relationship of sequence length with **P**_min _for this data, a line was fitted (significant r^2 ^value = 0.1, P < 0.001). Then, the intercept of this line was decreased until just 10% of protein sequences had CB regions picked for inclusion in the data set.

So that the smallest sequences do not have unreasonably high threshold values, the **P**_min _value was calculated at which 10% of all of the protein sequences in a proteome would have a CB region assigned to them. This second sequence-length-independent threshold **P**_min _value was used, where it was smaller than the sequence-length-dependent value. Using percentages of sequences in the range 5% to 15% to calculate these threshold **P**_min _values does not qualitatively change the main observations reported in the paper.

### CB signatures

All regions that have the same CB signature were grouped together. To allow for small differences in the order of recruitment to longer CB signatures, in some cases, we also analysed permutations of CB signatures (*e.g*., {*xzy*} and {*xyz*} are such permutations).

### Sequence annotations

Annotation of protein disorder was performed using DISOPRED [12], using default parameters trained to give a 5% false positive rate. The total fraction of predicted protein disorder in a CB region is given by the ***D ***value. Coiled coils were identified with the program MULTICOIL [17], using default parameters. Known protein domains were assigned using the ASTRAL 40% identity protein domain sequence set, and BLAST using e-value ≤ 0.01 [13,18]. Types of biased region that map to repetitive Zinc-finger-containing proteins (> 0.5 of the length of the protein) were numerous and were additionally filtered out.

GO (Gene Ontology; [19]) functional categories were taken from the annotation files provided on the Ensembl [16] and Gene Ontology [20] websites. Further GO term annotations were derived by mapping functional GO annotations for the PDB (downloaded from [20]) onto Ensembl protein annotations, using 50% sequence identity and 0.8 fractional sequence coverage (for the protein domain) as thresholds, using alignment made by the program BLASTP (e-value ≤ 0.0001) [13]. These thresholds were benchmarked on the complete SCOP protein domain sequence database [18], to give a 2% false positive rate for GO term transfer. Significant associations between GO terms and lists of protein sequences we calculated using binomial statistics, and a P'-value threshold of 0.05, where P' has been adjusted to account for multiple hypothesis testing, using the Bonferroni correction. In addition we used two functional supercategories, wherein all transcription-associated and non-transcription-associated GO terms were pooled together. The transcription-associated GO terms are: *GO:0006355; GO:0006357; GO:0006366; GO:0006367;GO:0016563;GO:0003676;GO:0003677;GO:0003700;GO:0003702;GO:0003704;GO:0003713;GO:0030374;GO:0030528*.

### Orthologs for conservation

Orthologs were calculated using the bidirectional best hits method and a BLASTP threshold of e-value ≤ 0.0001 [13], with the additional requirement for both of the potential orthologs to match each other over 0.6 of their sequence lengths. Potential orthologs were calculated both with and without the CB region masked, to give 'upper' and 'lower' bounds for ortholog detection.

## Abbreviations

LPS: Lowest Probability Subsequence; CB: compositional bias *or *compositionally-biased; GO: Gene Ontology.

## Authors' contributions

P.H. performed this work and wrote the paper.
